# Conditional punishment is a double-edged sword in promoting cooperation

**DOI:** 10.1038/s41598-017-18727-7

**Published:** 2018-01-11

**Authors:** Feng Huang, Xiaojie Chen, Long Wang

**Affiliations:** 1 Center for Systems and Control, College of Engineering, Peking University, Beijing, 100871 China; 2 School of Mathematical Sciences, University of Electronic Science and Technology of China, Chengdu, 611731 China

## Abstract

Punishment is widely recognized as an effective approach for averting from exploitation by free-riders in human society. However, punishment is costly, and thus rational individuals are unwilling to take the punishing action, resulting in the second-order free-rider problem. Recent experimental study evidences that individuals prefer conditional punishment, and their punishing decision depends on other members’ punishing decisions. In this work, we thus propose a theoretical model for conditional punishment and investigate how such conditional punishment influences cooperation in the public goods game. Considering conditional punishers only take the punishing action when the number of unconditional punishers exceeds a threshold number, we demonstrate that such conditional punishment induces the effect of a double-edged sword on the evolution of cooperation both in well-mixed and structured populations. Specifically, when it is relatively easy for conditional punishers to engage in the punishment activity corresponding to a low threshold value, cooperation can be promoted in comparison with the case without conditional punishment. Whereas when it is relatively difficult for conditional punishers to engage in the punishment activity corresponding to a high threshold value, cooperation is inhibited in comparison with the case without conditional punishment. Moreover, we verify that such double-edged sword effect exists in a wide range of model parameters and can be still observed in other different punishment regimes.

## Introduction

The solutions to many challenges in human societies, such as the management of public resources^1–3^ and the global warming^4–6^, all boil down to resort to a broad collective action of cooperation. However, the dilemma of helping others at a cost to ourselves or refraining from doing so but still profiting from the efforts provided by others^7^, always leads to the collapse of cooperation. As a solution to the dilemma of cooperation, costly punishment has attracted much attention both from the theoretical^8–11^ and experimental^12–17^ perspectives. But its side effect that the enforcement can lower the income of punishers is also highlighted^9,18^. Hence, whether contributing to the punishment pool or not becomes a similar dilemma as whether contributing to the public good or not^19–21^.

The puzzle about the emergence of costly punishment can be solved by considering some additional factors, such as reputation^22–24^, group selection^25–28^, social exclusion^29,30^, and consideration of sanctioning the second-order free-riders^10,31^. In addition, by including a loner strategy, voluntary participation also paves the way to solve the dilemma of costly punishment^8,9,32,33^. Based on the assumption that punishment is considered to be unconditional and uncoordinated individual action automatically triggered by defectors^34^, however, it seems that the loner strategy does not effectively address the inherent dilemma for the initial emergence of costly punishment, since rare punishers must undertake enough punishment when defection are prevalent^18^. On the contrary, the coordinated effort among punishers is well documented both in ethnographic evidence and behavioral experiments with communication or with the option of coordinating behavior^35,36^. It seems that such coordinated strategy can provide another method to overcome the problem of costly punishment because punishers do not bear the cost of punishment permanently. Motivated by the ethnographic evidence and behavioral experiments, a theoretically seminal work on coordinated punishment shows that cooperation can be sustained and such punishment can proliferate when rare^34^. Moreover, some other variants of punishment, such as conditional punishment^37^ defined by imposing a fine with a strength proportional to the number of punishers in their own groups and probabilistic sharing of punishment responsibility^38^, also play an important role in solving the problem of second-order free-riders.

It is worth mentioning that a recent behavior experiment found that individual’s punishing decision is on average significantly positively proportional to other members’ punishing decisions^39^. Actually, such sheep-flock effect of punishing behavior or the threshold effect of collective action^40^, is very ubiquitous in human societies and in animals. For example, when robbers implement a robbery in a public place, policemen may behave righteously and bring the thief to justice at once. While general civilians may hesitate to engage in sanction and their punishing decisions to robbers should significantly depend on the number of individuals who perform the punishment. And this novel behavior among punishers is completely distinct from the coordinated punishment investigated in some aforementioned works^34,37,38^. Hence, it still remains unclear how such conditional punishment, under which whether to sanction free-riders or not depends on the number of unconditional punishers in the group, influences the evolution of cooperation.

In this work, we then propose a theoretical model for conditional punishment in the context of public goods games, and consider that conditional punishers will participate in the punishment activity with other unconditional punishers only when the number of unconditional punishers in the group is not less than a threshold number, otherwise they will just cooperate. In addition to the consideration of well-mixed populations, we also investigate the conditional punishment in structured populations out of the interest for dynamics in some real social systems^41,42^. Considering that very little work has addressed questions about the relative efficacy of different types of punishment pointed out in ref.^17^, we further take into account different punishment forms^10,29,38^. As we will show in what follows, the introduction of conditional punishment induces the effect of a double-edged sword on the evolution of cooperation. That is, if the threshold for the number of unconditional punishers is low, more conditional punishers will jump on the bandwagon and punish free-riders, which sustains cooperation. Otherwise, a high threshold exacerbates the second-order free-rider problem of punishment. And we verify that such effect is robust against population structures and punishment regimes.

## Model

We consider that individuals in a population play the public goods game in which *G* individuals are chosen randomly to form a group for playing the game. Each player is set as a pure cooperator (C), a pure defector (D), an unconditional punisher (P), or a conditional punisher (M). Except for defectors who contribute nothing to the common pool but exploit others’ efforts, all three other strategists contribute a fixed amount *c* to the common pool. The sum of all contributions in the group will be multiplied by a synergy factor *r*, and then allotted equally among all group members irrespective of their contributions.

Subsequently, the punishment mechanism will work as long as there exists at least one defector and one punisher in the group. Each unconditional punisher will impose a fine *α* on each defector in the group. While all pure cooperators only contribute to the public good but refrain from punishing defectors, who are the second-order free-riders^18,43^. Conditional punishers are principally cooperators who contribute to the common pool, but meanwhile permanently observe the choices of other players in the group at an additional cost of *γ*. Such observation will assist conditional punishers to discern the number of unconditional punishers in the group. When the number of unconditional punishers is not less than the threshold *H*, which should be satisfied 0 < *H* < *G*, the punishing action from conditional punishers will be triggered. Each conditional punisher will impose the same fine *α* on each defector as a reaction. Otherwise, they do nothing but cooperate. Thus, in our model the punishing decision of conditional punishers to a defector significantly relies on the number of unconditional punishers. And when *H* is low, it means that it is relatively easy for conditional punishers to participate in the punishment activity. While when *H* is high, it means that the environment for conditional punishers to participate in the punishment activity is more harsh. In addition, each defector penalized for free-riding will bring a cost *β* to the community of punishers. And the associated costs are equally shared among individuals who participate in the punishment activity following a previous work^38^.

Accordingly, we designate the number of pure cooperators, pure defectors, unconditional punishers, and conditional punishers as *N*_*C*_, *N*_*D*_, *N*_*P*_, and *N*_*M*_ among the other *G* − 1 players in the group, respectively. And hence the payoffs of cooperators (Π_*C*_), defectors (Π_*D*_), unconditional punishers (Π_*P*_), and conditional punishers (Π_*M*_) from the group are given by, respectively,

1 $$\begin{array}{ll}{{\rm{\Pi }}}_{C} & =\,\frac{r(G-{N}_{D})c}{G}-c,\\ {{\rm{\Pi }}}_{D} & =\,\frac{r(G-{N}_{D}-\mathrm{1)}c}{G}-[{N}_{P}+\delta ({N}_{P}-H){N}_{M}]\alpha ,\\ {{\rm{\Pi }}}_{P} & =\,{{\rm{\Pi }}}_{C}-\frac{{N}_{D}\beta }{{N}_{P}+\delta ({N}_{p}+1-H){N}_{M}+1},\\ {\Pi }_{M} & =\,{{\rm{\Pi }}}_{C}-\delta ({N}_{P}-H)\frac{{N}_{D}\beta }{{N}_{P}+{N}_{M}+1}-\gamma ,\end{array}$$

where *δ*(*u*) is the Heaviside step function: *δ*(*u*) = 1 if *u* ≥ 0, otherwise *δ*(*u*) = 0. For the sake of comparison with the case of a structured population, we assume that the group size is *G* = 5 in this paper. Furthermore, without loss of generality, the contribution to the public good is considered to be *c* = 1. And to adhere to the existence of social dilemma^11,44,45^, the interval of *r* values is constrained as 1 < *r* < *G*.

As we have already defined, it is a key point that conditional punishers employ a more sophisticated strategy with following the trend, which characterizes the sheep-flock effect of punishing behavior. More specifically, such a player only behaves as a pure cooperator and refuses to engage in punishment if the number of unconditional punishers is less than a critical threshold *H*. Otherwise, they will undertake the obligation of punishing defectors, who play the role of unconditional punishers. Such propensities of following the trend for conditional punishers are characterized via the *δ* function. In general, the value of *H* can characterize the level of willingness or difficulty for conditional punishers engaging in punishment. Thus, the threshold *H* is a key parameter in our model. In what follows, we will present the evolutionary dynamics both in well-mixed and structured populations for low and high values of *H*. In particular, we will show the effects of conditional punishment on the evolution of cooperation by comparing with the case in which conditional punishment is not introduced.

## Results

### Infinite well-mixed populations

**Figure 1:**
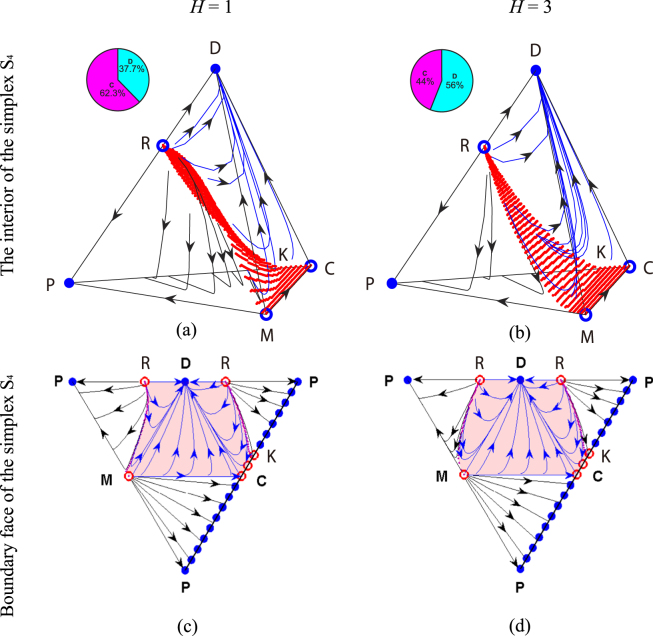
Flow diagrams in the interior of the simplex *S*_4_ and on its boundary faces based on replicator dynamics. Top row (bottom row) depicts evolutionary dynamics in the interior (on the boundary faces) of the simplex *S*_4_ for *H* = 1 (panels (**a**) and (**c**)) and *H* = 3 (panels (**b**) and (**d**)), respectively. Stable fixed points are depicted with solid blue circles, while unstable fixed points are depicted with open blue or red circles. Arrows indicate the direction of evolution. In the interior of the simplex *S*_4_ and on its boundary faces, the system will evolve to the stable equilibrium—either all defectors (vertex D) or the coexistence of cooperators and unconditional punishers (segment PK), in dependence on the initial conditions. The red surface separates the basins of attraction for vertex D and segment PK. In each pie chart, the likelihood that the equilibrium segment PK evolves is indicated by red, and green for the likelihood that the equilibrium D evolves. Additionally, the light red domain in the second row represents the attractive basin of defection on the boundary faces of the simplex *S*_4_. Parameters in panels (**a**) and (**c**): *r* = 3, *c* = 1, *G* = 5, *α* = 1.0, *β* = 0.7, *γ* = 0.05, and *H* = 1. Parameters in panels (**b**) and (**d**): *r* = 3, *c* = 1, *G* = 5, *α* = 1.0, *β* = 0.7, *γ* = 0.05, and *H* = 3.

Based on replicator equations, we first present the evolutionary dynamics in infinite well-mixed populations. In Fig. 1, the flow diagrams are shown in the interior of the simplex *S*_4_ and on its boundary faces for two different threshold values, respectively. We find that when conditional punishment is considered, the system will evolve to either the state of all defectors (vertex D) or the coexistence state of cooperators and unconditional punishers (segment PK), no matter whether the threshold value is low or high (Fig. 1(a) and (b)). And such evolutionary outcomes are not changed in comparison with the case in which conditional punishment is not introduced (see the triangle PDC in Fig. 1(c) and (d)). In the simplex *S*_4_, accordingly there exists a surface which divides the whole strategy state space into two basins of attraction. In particular, the unstable interior equilibrium R on the edge DP can be determined by the real root z^*^∈(0, 1) of the function *g*(*z*) = *β*{(1)/(*z*)[(1 − *z*)^*G*^ − 1] + (*α*(*G *− 1)*z*)/(*β*) + (*rc*)/(*Gβ*) − (*c*)/(*β*) + 1} (Methods for infinite populations).

Furthermore, we analyze the basin of attraction in the simplex *S*_4_ by numerical calculations, as shown in the pie chart of Fig. 1. We find that for low *H* = 1, the cooperative basin of attraction occupies 62.3% of the whole strategy state space in the simplex *S*_4_. While for high *H* = 3, it only occupies 44.0% of the whole strategy state space. It indicates that the cooperative basin of attraction decreases with increasing the threshold value *H*. On the other hand, we note that the cooperative basin of attraction occupies 51.5% of the whole triangle PDC shown in Fig. 1(c) and (d), which corresponds to the case without conditional punishment. Thus, we can conclude that the introduction of conditional punishment induces a double-edge sword effect on cooperation. That is, when it is easy for conditional punishers to participate in the punishment activity (low threshold value), cooperation is promoted in comparison with the case in which conditional punishment is not introduced. While when it is difficult for conditional punishers to participate in the punishment activity (high threshold value), cooperation is inhibited in comparison with the same case where conditional punishment is not introduced.

**Figure 2:**
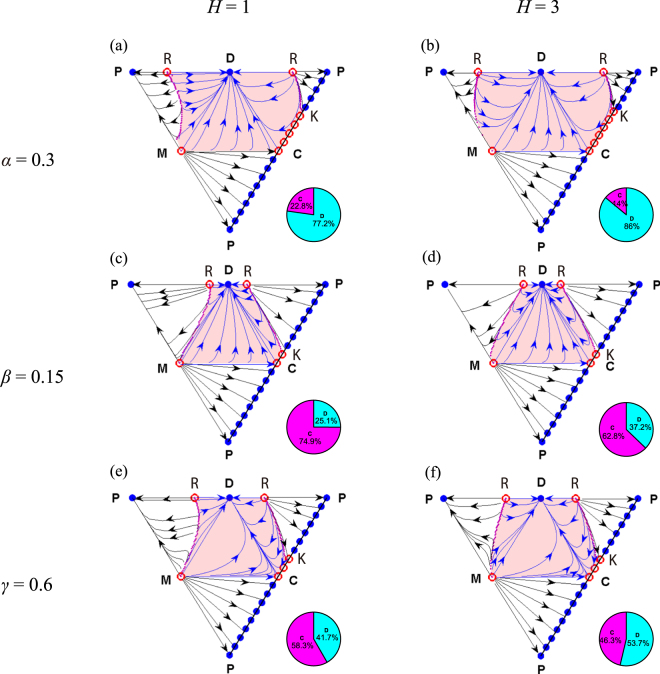
Evolution on the boundary faces of the simplex *S*_4_ for different model parameters. Unstable equilibria are indicated by open red circles, and stable equilibria are indicated by solid blue circles. The defection basin of attraction is depicted by the light red shadow, and the remaining domain is the ‘cooperative’ basin of attraction. In each pie chart, the red domain represents the likelihood that the equilibrium segment PK evolves, and the green domain for the likelihood that the equilibrium D evolves in the interior of the simplex *S*_4_. For the cases without the strategy of conditional punisher, the cooperative basin of attraction occupies 16.4% ((**a**) and (**b**)), 69.1% ((**c**) and (**d**)), and 48.4% ((**e**) and (**f**)) of the whole triangle DCP, respectively. Parameters: *r* = 3, *c* = 1, *G* = 5, *α* = 0.3, *β* = 0.7, and *γ* = 0.05 in (**a**) and (**b**); *r* = 3, *c* = 1, *G* = 5, *α* = 1.0, *β* = 0.15, and *γ* = 0.05 in (**c**) and (**d**); *r* = 3, *c* = 1, *G* = 5, *α* = 1.0, *β* = 0.7, and *γ* = 0.6 in (**e**) and (**f**).

By respectively changing the model parameters (*α*, *β*, and *γ*), we show the evolution of strategies on the boundary faces of the simplex *S*_4_ again (Fig. 2). It is found that the stability of the system does not change when the parameter values are properly altered. And in comparison with Fig. 1, we further find that the cooperative basin of attraction decreases with decreasing the *α* value, or increasing the *β* value, no matter whether the threshold value *H* is low or high. Moreover, increasing the *γ* value for low *H* also decreases the cooperative basin of attraction. But this effect reverses for high *H*. Importantly, we still see that the double-edge sword effect exists even if these parameter values are changed significantly, which indicates that this finding of the double-edge sword effect remains valid in a broad range of model parameters.

### Finite well-mixed populations

**Figure 3:**
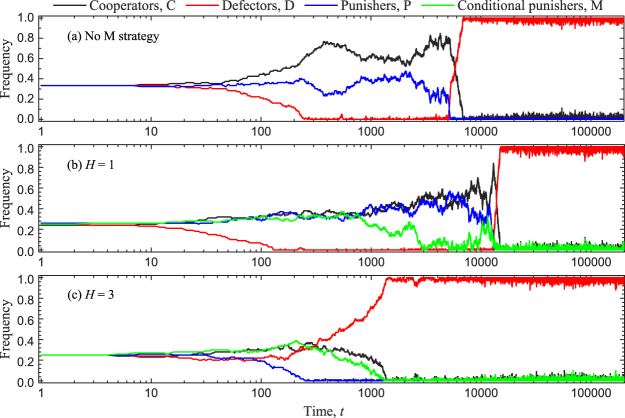
Time evolution of strategies for three different situations. Panel (**a**) shows the time evolution of all three strategies when conditional punishment is not considered. In the presence of conditional punisher strategy, panel (**b**) shows the time evolution of all four strategies for *H* = 1, but panel (**c**) for *H* = 3. Individual-based simulations run over 10^9^ time steps, and here we only present the outcomes for 2 × 10^5^ time steps. Parameters: *r* = 3, *c* = 1, *G* = 5, *N* = 100, *α* = 1.0, *β* = 0.7, *γ* = 0.05, *s* = 2.0, and *μ* = 0.001.

We continue to study the effects of conditional punishment on the evolution of cooperation in finite well-mixed populations. Based on the social learning dynamics^10^ with an arbitrary exploration rate *μ* (Methods for finite populations), we first show the time evolution of strategies for three different situations by individual-based simulations, as shown in Fig. 3. It is noted that for a relatively small threshold the population can temporarily evolve into a quasi-stable state^46^ where defectors are suppressed, and only cooperators and unconditional punishers coexist due to neutral drift. Although such quasi-equilibrium is not the evolutionarily stable state no matter whether the conditional punishment is introduced or not (Fig. 3(a) and (b)), the time duration of such quasi-equilibrium can be changed significantly with the change of threshold values once the conditional punishment is introduced. In comparison with the time duration of the quasi-stable state for no conditional punishment as shown in Fig. 3(a), the time duration of the quasi-stable state for low *H* = 1 is longer (Fig. 3(b)). While for high *H* = 3, the quasi-stable state almost does not emerge and the system rapidly evolves into the globally stable equilibrium where the whole population is taken over completely by defectors (Fig. 3(c)). This indicates that the introduction of conditional punishment can still induce a double-edged sword effect in finite well-mixed populations.

**Figure 4:**
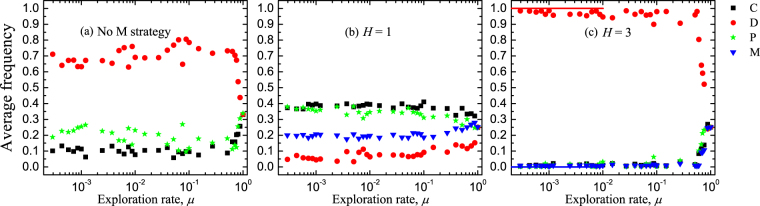
Average frequencies of strategies as a function of mutation rate in three different situations. Panel (**a**) shows the average frequencies of three strategies when conditional punishment is not considered. In the presence of conditional punisher strategy, panel (**b**) shows the average frequencies of all four strategies for *H* = 1, but panel (**c**) for *H* = 3. Symbols indicate results obtained from individual-based simulations (by averaging over 10^4^–10^5^ imitation steps for each player and by doing 50 independent runs) and solid lines indicate analytical approximations for very small values of *μ*. Parameters: *r* = 3, *c* = 1, *G* = 5, *N* = 100, *α* = 1.0, *β* = 0.7, *γ* = 0.05, *s* = 2.0, and *μ* = 0.001.

In order to illustrate the robustness of the double-edged sword effect in finite populations, we further present the average frequencies of strategies as a function of mutation rate, as shown in Fig. 4. The simulation results are indicated by data points, and the analytical approximations for very small values of *μ* are indicated by solid lines (Methods for finite populations). We find that for a sufficiently large *μ* (close to 1), random exploration dominates and results in roughly equal average frequencies for all available strategies. But for a small or moderate *μ*, the results can be significantly influenced by the threshold *H* in comparison with the results without the strategy of conditional punisher. Specifically, for low *H* = 1 the average frequencies of cooperators and (unconditional and conditional) punishers are higher than the frequency of defectors (Fig. 4(b)). And importantly, these frequencies are much higher than those in the case where the conditional punisher strategy is not considered, as shown in Fig. 4(a). This shows that cooperation is promoted for low threshold values when conditional punisher strategy is introduced. However, for high *H* = 3 the average frequencies of cooperators and (unconditional and conditional) punishers are much lower than the frequency of defectors (Fig. 4(c)). Correspondingly, these frequencies are also lower than the cooperators’ and punishers’ frequencies in the case without conditional punisher strategy. This indicates that cooperation is inhibited for high threshold values when conditional punisher strategy is introduced. Therefore, the double-edged sword effect still exists in a broad range of *μ* in finite well-mixed populations.

### Structured populations

In contrast to the well-mixed case, the fact that the interactions among players are not typically random but rather that each player merely interacts with a set of fixed neighbors in the population^38,47–50^, is taken into account in structured populations. Usually, it can lead to some novel and counterintuitive results which are absent in a well-mixed population^51^.

**Figure 5:**
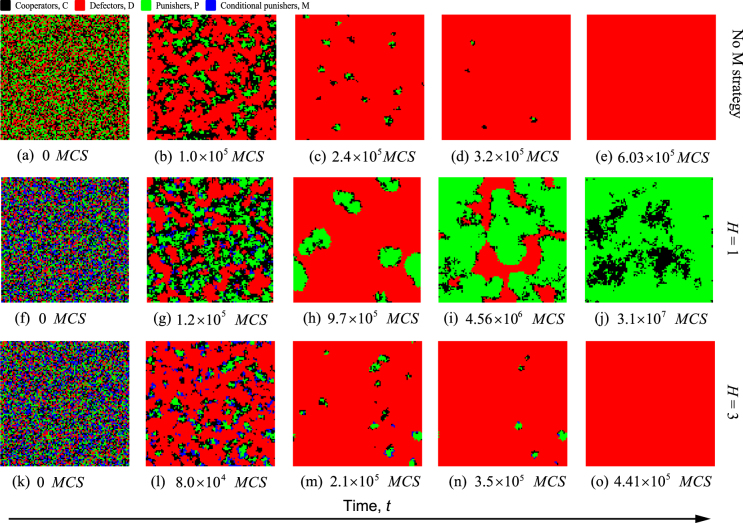
Spatial patterns of strategies over time for three different situations. Cooperators (C) are denoted by black, defectors (D) by red, unconditional punishers (P) by green, and conditional punishers (M) by blue. Top row depicts the typical snapshots over time in the case where the strategy of conditional punisher is not considered. Middle row depicts the typical snapshots over time in the case where the strategy of conditional punisher is introduced and the threshold value is *H* = 1, but *H* = 3 for bottom row. The times of strategy update by Monte Carlo simulation are also shown for each snapshot. Parameters: *G* = 5, *r* = 2.0, *α* = 1.0, *β* = 0.8, *c* = 1, *γ* = 0.05, *s* = 2, and *L* = 100.

To explore the effects of conditional punishment on cooperation in structured populations, here we show a series of snapshots on a square lattice with the von Neumann neighborhood to depict the spatial formation of all strategies over time (Methods for structured populations), as shown in Fig. 5. First, some characteristic snapshots of the spatial formation are presented in the case where conditional punishment is not considered (top row of Fig. 5). We find that defectors can quickly fight against other two strategists, which results in that cooperators or unconditional punishers can only form small tiny clusters. With the invasion of defectors, these small clusters formed by cooperative individuals finally disappear completely, which leaves defectors to take over the population. Nevertheless, when conditional punishment is considered and the threshold value is low (middle row of Fig. 5), defectors only have some competitive advantages over the other three strategists during the initial period of the evolution, and they can then utilize these advantages to rapidly invade the whole population. Ultimately, it results in the extinction of conditional punishers as well as the decrease of cooperators and unconditional punishers. But when cooperators and unconditional punishers form the compact clusters, they can reverse the invasion of defectors and expand across the whole population. Such results are indicated by the spatial formation that the isolated islands of defectors (depicted by red) are in the sea of cooperators (depicted by black) and punishers (depicted by green), and finally disappear completely. On the contrary, when the threshold *H* is high (bottom row of Fig. 5), the negative effect of conditional punishers on cooperation is highlighted. Because conditional punishers have less chances to participate in the punishment activity with unconditional punishers, this immediately provides a chance for defectors to obtain a relatively higher fitness. Consequently, defectors can permanently remain successful in the structured public goods game. Hence, in structured populations, the effect of conditional punishment on the evolution of cooperation is still a double-edged sword. When conditional punishment is not considered in the public goods game, the population evolves towards a homogenous state of full defectors (D-only phase), finally. However, if the strategy of conditional punisher is considered, a low threshold value will drive the population to evolve into the coexistence of cooperators and unconditional punishers (a mixed C + P phase), which shows the positive effect of conditional punishment on cooperation. But if a high threshold value is applied, the system will transform from the mixed C + P phase to a D-only phase, similar to the previous finding in spatial public goods game with four strategies^38,51,52^. And it takes a shorter time to reach the D-only phase in comparison with the case without conditional punishment, which shows the negative effect of conditional punishment on cooperation.

**Figure 6:**
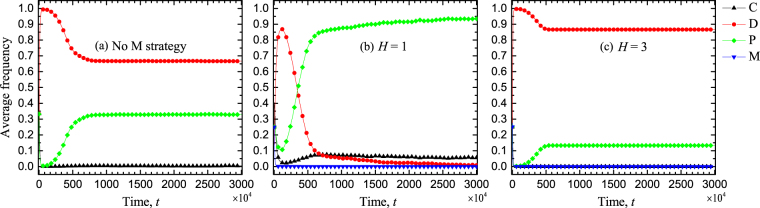
Time evolution of average frequencies of strategies by averaging 30 independent runs in structured populations. Panel (**a**) depicts the average frequencies of the three strategies over time in the case where the strategy of conditional punisher is not considered. Panel (**b**) depicts the average frequencies of all four strategies over time in the case where the strategy of conditional punisher is introduced and the threshold value is *H* = 1, but *H* = 3 for panel (**c**). Parameters: *G* = 5, *r* = 2.0, *α* = 1.0, *β* = 0.8, *c* = 1, *γ* = 0.05, *s* = 2, and *L* = 100.

In order to eliminate the influence of randomness on the evolutionary outcomes, we further calculate the average frequencies of strategies over time by doing 30 independent runs for those three different situations studied in Fig. 5, as illustrated in Fig. 6. It is our goal to verify the existence of the double-edged sword effect in structured populations by evaluating the average frequencies of strategies in equilibrium. We find that when there is no conditional punishers engaging in structured public goods game, the average frequency of cooperators in equilibrium is zero. And the average frequency of punishers in equilibrium is about 0.33, which is much smaller than that of defectors whose frequency is about 0.67 (Fig. 6(a)). Whereas when conditional punishment is considered and the threshold value is low, we see that cooperators and unconditional punishers coexist in equilibrium and the average frequencies of the other two strategies converge to zero (Fig. 6(b)). It implies that the average level of cooperation is significantly increased in comparison with the case without conditional punishment. On the contrary, when the threshold value is high, we observe the similar results to the case without conditional punishment. But the average frequency of unconditional punishers in equilibrium is about 0.15 (Fig. 6(c)), which is much lower than that in the case without conditional punishment. This shows that cooperation is obviously inhibited when it compares with the results in the case without conditional punishment. We thus conclude that the double-edged sword effect is also embodied in structured populations. In addition, we have checked that such effect still exists even if we properly change the initial conditions for these three situations.

## Discussion

Since a decision-making may cause damage to our own interests, the reaction made by depending on others’ decisions is usually a dominant strategy most of the time. The effect of sheep-flock in our life is a typical example. Unlike the definition given by the previous study^37^, our model regarding conditional punishment characterizes the sheep-flock effect of the punishing behavior which has been documented in the experimental research^39^. By means of theoretical analysis and computer simulations, we have explored the effects of conditional punishment on the evolution of cooperation. Conceptually similar to the conditional cooperation or conditional participation in joint efforts^7,51,53,54^, conditional punishers can utilize the advantages of both unconditional cooperators and unconditional punishers. But simultaneously, it also induces the effect of a double-edged sword on cooperation. When it is relatively easy for conditional punishers to participate in the punishment activity, the invasion of defectors can be controlled by punishment, and the cost caused by punishment can be also shared by more individuals. Thus, in comparison with the case without conditional punishment, cooperation can be further promoted. Whereas when it is relatively difficult for conditional punishers to participate in punishment activity, they are more willing to perform prosocial cooperation rather than spiteful punishment. In this way, the threat of sanctioning free-riders is so weak that the environment for cooperation to thrive becomes more harsh. Thus, cooperation is inhibited when it compares with the outcome in the case without conditional punishment.

Moreover, it is necessary to point out that in our model the ability to recognize the level of unconditional punishers is not self-serving but costly for conditional punishers, because the self-serving function does not seem to be the feature of punishment in real life^29,51^. Accordingly, the extra cost makes conditional punishers do not have the competitive advantages over other two cooperative strategists, no matter what the threshold value is. And meanwhile it also differentiates the strategy of conditional punisher from other cooperative strategies, essentially. Our study thus reveals the significant role of additional cost in the evolution of cooperation, and shows that the introduction of conditional punishment can alleviate or exacerbate the second-order free-rider problem^9,29,55^, which strongly depends on the the threshold value. Consequently, conditional punishment induces the effect of a double-edged sword on the evolution of cooperation.

Although different punishment modes can result in different outcomes according to previous studies^10,17^, we verify that the double-edged sword effect found in our model is also valid for other punishment regimes, such as peer punishment and a variant of its (see Supplementary Information). Undeniably, in the framework of our model, sanction is merely targeted at free-riders, and the possibility of anti-social punishment^24,56,57^ that non-cooperators attack cooperators is excluded a priori. To make up this deficiency, we additionally consider a model variant that includes the possibility of anti-social punishment (see Supplementary Information). When defectors suffer from the sanction of punishers, it will trigger defectors to revenge all members in the group. Surprisingly, cooperation is still sustained in the population. In addition, as an important direction to develop our model, considering the heterogeneity of the threshold^52,58,59^ for matching the reality well is worth the effort in the future.

## Methods

### Evolutionary dynamics in infinite well-mixed populations

We study the evolution of strategies in infinite well-mixed populations based on replicator dynamics^60,61^. First, we define that the fraction of cooperators (C), defectors (D), unconditional punishers (P), and conditional punishers (M) can be denoted by *x*, *y*, *z*, and *w*, respectively. Thus we have *x* + *y* + *z* + *w* = 1. Accordingly, the replicator equations are given by

2 $$\begin{array}{rcl}\dot{x} & = & x({P}_{C}-\bar{P}),\\ \dot{y} & = & y({P}_{D}-\bar{P}),\\ \dot{z} & = & z({P}_{P}-\bar{P}),\end{array}$$

where dots denote the derivatives with respect to time *t* and *P*_*i*_ designates the expected payoff for each strategy *i* (*i* = C, D, P, or M), which is given by

3 $${P}_{i}=\sum _{0\le {N}_{s}\le G-1}\frac{(G-\mathrm{1)!}}{{N}_{C}!{N}_{D}!{N}_{P}!{N}_{M}!}{x}^{{N}_{C}}{y}^{{N}_{D}}{z}^{{N}_{P}}{w}^{{N}_{M}}{{\rm{\Pi }}}_{i},$$

where *N*_*s*_ is the number of players choosing strategy *s* (*s* = *C*, *D*, *P*, *M*) in a group, hence ∑_*s*_*N*_*s*_ = *G* − 1. Π_*i*_ represents the payoff of strategy *i*, which is defined by Eq. (1). $\bar{P}$ describes the average payoff of the entire population, which is given by $\bar{P}=x{P}_{C}+y{P}_{D}+z{P}_{P}+w{P}_{M}$.

For discussing the evolution of these four strategies, we first consider there are no any punishers in the population. In this way, defectors can exploit the effort of cooperators permanently. Therefore, natural selection will always favor defectors to take over the population, irrespective of the initial conditions.

However, the introduction of punishers can effectively reverse the negative situation. Thus we consider that only defectors and unconditional punishers are presented in the population, namely *y* + *z* = 1. Then the replicator equation degenerates to $\dot{z}=z\mathrm{(1}-z)({P}_{P}-{P}_{D})$. In this situation, the average payoff of punishers *P*_*P*_ is given by

4 $$\begin{array}{ll}{P}_{P} & =\,\sum _{k=0}^{G-1}(\begin{array}{c}G-1\\ k\end{array}){z}^{k}{\mathrm{(1}-z)}^{G-k-1}[\frac{r(k+\mathrm{1)}c}{G}-c-\frac{(G-k-\mathrm{1)}\beta }{k+1}]\\  & =\,\frac{\beta }{z}[{\mathrm{(1}-z)}^{G}-1]+\frac{rc}{G}[(G-\mathrm{1)}z+1]+\beta -c\mathrm{.}\end{array}$$

Similarly, the average payoff of defectors *P*_*D*_ is given by

5 $$\begin{array}{ll}{P}_{D} & =\,\sum _{k=0}^{G-1}(\begin{array}{c}G-1\\ k\end{array}){z}^{k}{\mathrm{(1}-z)}^{G-k-1}[\frac{rkc}{G}-k\alpha ]\\  & =\,(\frac{rc}{G}-\alpha )(G-\mathrm{1)}z\mathrm{.}\end{array}$$

With these expressions, the replicator equation has two boundary equilibria, namely *z* = 0 and *z* = 1. On the other hand, the interior equilibria can be determined by the roots of the function *g*(*z*) := *P*_*P*_ − *P*_*D*_, thus obtaining

6 $$g(z)=\frac{\beta }{z}\mathrm{[(1}-z{)}^{G}-\mathrm{1]}+\alpha (G-\mathrm{1)}z+\frac{rc}{G}+\beta -c\mathrm{.}$$

It follows that $g\mathrm{(0)}={\mathrm{lim}}_{z\to {0}^{+}}g(z)=\frac{rc}{G}-c+\beta \mathrm{(1}-G) < 0$ with 1 < *r* < *G* and *c* = 1. Note that the function *g*(*z*) can be approximated by *g*(*z*) ≈ (*α* + *Gβ*/2)(*G* − 1)*z* + *β*(1 − *G*) + (*rc*)/(*G*) − *c*. Thus the function *g*(*z*) is strictly increasing since *g*′(*z*) ≈ (*α* + *Gβ*/2)(*G* − 1) > 0. Accordingly, the interior equilibrium is determined by *g*(1) = *α*(*G* − 1) + (*rc*)/(*G*) − *c*, from which we have the following two conclusions:

1. When *α* > ((*G* − *r*)*c*)/(*G*(*G* − 1)), the replicator equation has only one interior equilibrium z^*^∈(0, 1), but it is unstable since *g*′(z^*^) > 0. The two boundary equilibria *z* = 0 and *z* = 1 are both stable.
2. When *α* ≤ ((*G* − *r*)*c*)/(*G*(*G* − 1)), the replicator equation has no interior equilibria in (0, 1). *z* = 0 is a stable equilibrium, while *z* = 1 is an unstable equilibrium.

Moreover, if there are no defectors in the population, the average payoff of cooperators is equal to that the unconditional punishers obtain from the public goods game. And it is higher than the average payoff of conditional punishers, because the latter have to pay the observation cost. Thus natural selection will support the system to evolve into the coexistence state of cooperators and punishers because of neutral drift.

### Evolutionary dynamics in finite well-mixed populations

We denote that the population of size *N* contains *X* cooperators, *Y* defectors, *Z* unconditional punishers, and *W* conditional punishers. Thus we have *X* + *Y* + *Z* + *W* = *N*, and the average payoffs of cooperators (C), defectors (D), unconditional punishers (P), and conditional punishers (M) can be given by, respectively,

7 $$\begin{array}{ll}{P}_{C} & =\,\sum _{k=0}^{G-1}\frac{(\begin{array}{c}N-1-Y\\ k\end{array})(\begin{array}{c}Y\\ G-k-1\end{array})}{(\begin{array}{c}N-1\\ G-1\end{array})}[\frac{r(k+\mathrm{1)}c}{G}-c]\\  & =\,\frac{rc}{G}[\frac{(G-\mathrm{1)(}N-Y-\mathrm{1)}}{N-1}+1]-c,\end{array}$$

8 $${P}_{D}=\sum _{k\mathrm{=0}}^{G-1}\frac{(\begin{array}{c}N-Y\\ k\end{array})(\begin{array}{c}Y-1\\ N-k-1\end{array})}{(\begin{array}{c}N-1\\ G-1\end{array})}\frac{rkc}{G}-\sum _{0\le l,m\le G-1}\frac{(\begin{array}{c}Z\\ l\end{array})(\begin{array}{c}W\\ m\end{array})(\begin{array}{c}N-Z-W-1\\ G-l-m-1\end{array})}{(\begin{array}{c}N-1\\ G-1\end{array})}[l+\delta (l-H)m]\alpha ,$$

9 $${P}_{P}={P}_{C}-\sum _{0\le l,m,n\le G-1}\frac{(\begin{array}{c}Z-1\\ l\end{array})(\begin{array}{c}W\\ m\end{array})(\begin{array}{c}Y\\ n\end{array})(\begin{array}{c}N-Z-W-Y\\ G-l-m-n-1\end{array})}{(\begin{array}{c}N-1\\ G-1\end{array})}\frac{n\beta }{l+1+\delta (l+1-H)m},$$

and

10 $${P}_{M}={P}_{C}-\gamma -\sum _{0\le l,m,n\le G-1}\frac{(\begin{array}{c}Z\\ l\end{array})(\begin{array}{c}W-1\\ m\end{array})(\begin{array}{c}Y\\ n\end{array})(\begin{array}{c}N-Z-W-Y\\ G-l-m-n-1\end{array})}{(\begin{array}{c}N-1\\ G-1\end{array})}\delta (l-H)\frac{n\beta }{l+m+1},$$

where *k*, *l*, *m*, and *n* represent the number of contributors, unconditional punishers, conditional punishers, and defectors among *G* − 1 players in a group, respectively.

Next, we employ a so-called social learning process^10^ to describe the evolution of all strategies in finite well-mixed populations. Let us denote that *P*_*u*_ and *P*_*v*_ are the average payoffs of two randomly chosen players *u* and *v*, respectively. Under pairwise comparison rule^45,62,63^, player *u* adopts the strategy of player *v* with a probability given by the Fermi function^64^

11 $$q=\frac{1}{1+exp[-s({P}_{v}-{P}_{u})]},$$

where the imitation strength *s* ≥ 0 measures the intensity of selection that determines the level of uncertainty in the strategy imitation process^4,11^. Without loss of generality, we use a representative value *s* = 2^11,19,51^ in finite well-mixed and structured populations, which implies that the better performing players are readily imitated, but it is not impossible to adopt the strategy of a player performing worse.

Then we denote that *N*_*i*_ is the number of players choosing strategy *i*. Hence the probability that one chosen as a focal player out of *N*_*i*_ players with strategy *i* imitates another player of the *N*_*j*_ = *N* − *N*_*i*_ players with strategy *j* (*j* ≠ *i* and *j* = C, D, P, or M) is given by

12 $${\tau }_{i\to j}({N}_{i})=\frac{{N}_{i}}{N}\frac{N-{N}_{i}}{N}\frac{1}{1+exp[-s({P}_{j}-{P}_{i})]}\mathrm{.}$$

As a result, the fixation probability that characterizes the fixation of the dissident strategy *j* caused by imitation in the population can be computed by

13 $${\rho }_{ij}=\frac{1}{1+\sum _{q=1}^{N-1}\prod _{{N}_{j}=1}^{q}\frac{{\tau }_{j\to i}({N}_{j})}{{\tau }_{i\to j}({N}_{j})}}\mathrm{.}$$

It is noted that the equation *N*_*j*_ = *N* − *N*_*i*_ is always met, so the fixation probability *ρ*_*ij*_ can be simplified to

14 $${\rho }_{ij}=\frac{1}{1+\sum _{q\mathrm{=1}}^{N-1}exp[s\sum _{{N}_{j}\mathrm{=1}}^{q}({P}_{i}-{P}_{j})]}\mathrm{.}$$

Furthermore, let us denote that the homogeneous population with *N*_*i*_ = *N* is *All*_*i*_ and the random exploration rate is *μ*. In the case of four strategies (*C*, *D*, *P*, and *M*), with probability *μ*/3 a single individual randomly switches from strategy *i* to the strategy *j* (*j* ≠ *i*). Thus the transition probability *p*_*ij*_ from *All*_*i*_ to *All*_*j*_ is *μρ*_*ij*_/3. In this way, the transition matrix of the complete Markov chain can be written as *Pr* = [*p*_*ij*_]_4×4_. Accordingly, the stationary distribution which describes the percentage of time spent by the state of the population in the vicinity of the homogeneous state^10^, is given by the normalized left eigenvector to the eigenvalue 1. In addition, it is shown that the stationary distribution of the full system converges to the stationary distribution of this ‘embedded’ Markov chain on the homogeneous states^65,66^ for *μ* → 0, of which transition probabilities from *All*_*i*_ to *All*_*j*_(*j* ≠ *i*) are given by *ρ*_*ij*_/3^10^. Thus for four competitive strategies, the transition matrix can be concisely written by15where *j* is subject to three other strategies in the group except the imitator itself.

In particular, in the limiting case of strong imitation (*s* → +∞), the transition matrix can be significantly simplified by

16 $$Pr=(\begin{array}{llll}\frac{2N-1}{3N} & \frac{1}{3} & \frac{1}{3N} & 0\\ 0 & 1 & 0 & 0\\ \frac{1}{3N} & 0 & \frac{3N-1}{3N} & 0\\ \frac{1}{3} & \frac{1}{3} & \frac{1}{3} & 0\end{array}).$$

And the stationary distribution (the left eigenvector to the eigenvalue 1) is easily given by (0, 1, 0, 0), which implies the population becomes a stable regime of defectors, leading to the tragedy of the commons.

### Individual-based simulations for finite well-mixed populations

We consider a finite well-mixed population with a constant size *N*. Each individual achieves an expected payoff defined by Eqs (7)–(10) based on the random sampling of the interaction groups. Strategies evolve in dependence on a mutation-selection process defined in discrete time^7^. At each time step, a player *u* is randomly selected to update. With probability *μ*, the player *u* undergoes a mutation and randomly adopts one strategy from the space of available strategies. With probability 1 − *μ*, another individual *v* is randomly selected to act as a role model for player *u*. Then player *u* adopts the strategy of player *v* with a probability *q* defined by Eq. (11). Otherwise, player *u* sticks to its strategy with the probability 1 − *q*.

### Individual-based simulations for structured populations

Here, we consider a structured population where the public goods game is staged on a *L* × *L* square lattice with periodic boundary conditions. *L*^2^ players are arranged into overlapping groups of size *G* = 5 such that everyone is connected to its *G* − 1 nearest neighbors, which implies that each individual is involved in *G* different groups. Hence the overall payoffs for each player are the sum of all the profits acquired from *G* groups. Initially, the player on every site is designated either as a cooperator, defector, unconditional punisher, or conditional punisher with equal probability. At every time step, a player *u* is randomly selected to play the public goods game with its four neighbors as a member of all five groups and obtains its overall payoffs *P*_*u*_. Similarly, another player *v*, one of the four nearest neighbors, is chosen randomly and acquires its total payoffs *P*_*v*_ in the same way. If their strategies are different, the imitation is executed with the probability defined by Eq. (11). In each full round of the game, every player has one chance to imitate from one of their neighbors on average^19,37,51^.

## Electronic supplementary material

Supplementary Information
