# Supplementary material for: Conditional punishment is a double-edged sword in promoting cooperation
Source: Sci Rep. 2018 Jan 11;8:528. doi: 10.1038/s41598-017-18727-7 (PMC5764993; doi:10.1038/s41598-017-18727-7)
Supplement: Supplementary file 1 — Supplementary Information [file 41598_2017_18727_MOESM1_ESM.pdf]

Supplementary Information: Conditional punishment is a double-edged sword in promoting cooperation

Feng Huang[1,2], Xiaojie Chen[2,*], and Long Wang[1]

1 Center for Systems and Control, College of Engineering, Peking University, Beijing 100871, China
2 School of Mathematical Sciences, University of Electronic Science and Technology of China, Chengdu 611731, China
* xiaojiechen@uestc.edu.cn

In the Supplementary Information, we consider the conditional punisher strategy in the public goods game with peer punishment [1, 2] or a variant of peer punishment [3], and explore whether the effect of a double-edged sword induced by conditional punishment still exists when these two different punishment regimes are used instead of the punishment regime in the main text. In addition, to include the possibility of anti-social punishment, we also consider a variant model on the basis of our main model.

1 Other punishment regimes

1.1 Model description

In this section, based on previous works [1, 2, 3] we directly give the expressions of the payoffs for different strategies when the two different punishment regimes are considered into the public goods game, respectively. Accordingly, when peer punishment is considered, the payoffs of cooperators, defectors, unconditional punishers, and conditional punishers from one interaction group can be given by, respectively,

$$\begin{aligned}
\Pi_C &= \frac{r(G-N_D)c}{G} - c,\\
\Pi_D &= \frac{r(G-N_D-1)c}{G} - \left[N_P + \delta(N_P - H)N_M\right]\alpha,\\
\Pi_P &= \Pi_C - N_D\beta,\\
\Pi_M &= \Pi_C - \delta(N_P - H)N_D\beta - \gamma.
\end{aligned} \tag{1}$$

When the variant of peer punishment is considered, we assume that each defector in the group is punished with a fine α if at least one punisher is present, and punishers, on the other hand, equally share the associated costs following Ref. [3]. Accordingly, the payoffs of cooperators, defectors, unconditional punishers, and conditional

punishers from one group are given by, respectively,

$$\begin{aligned}
\Pi_C &= \frac{r(G-N_D)c}{G} - c, \\
\Pi_D &= \frac{r(G-N_D-1)c}{G} - \delta(N_P-1)\alpha, \\
\Pi_P &= \Pi_C - \frac{N_D\beta}{N_P+\delta(N_p+1-H)N_M+1}, \\
\Pi_M &= \Pi_C - \delta(N_P-H)\frac{N_D\beta}{N_P+N_M+1} - \gamma.
\end{aligned} \tag{2}$$

Based on the above models, in what follows, we investigate the effects of conditional punishment on the evolution of cooperation in well-mixed and structured populations, respectively.

1.2 Peer punishment

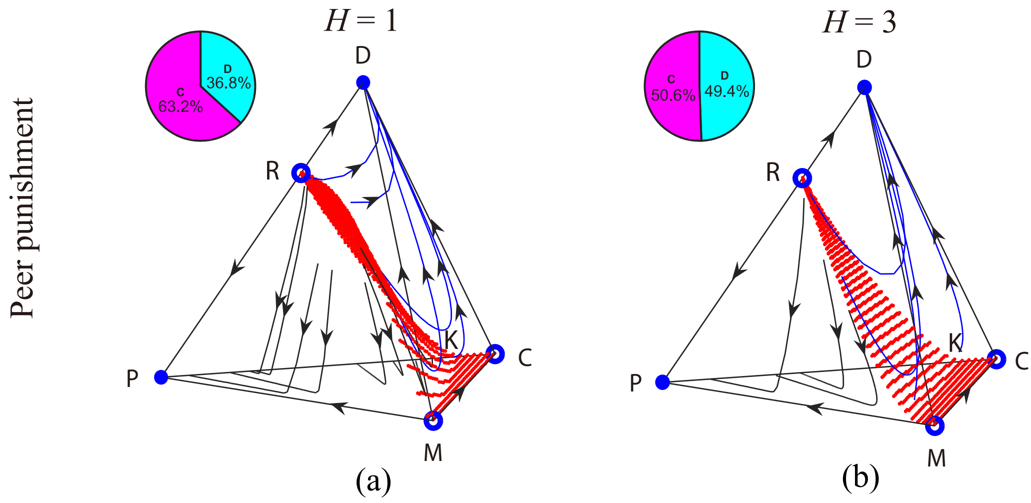

Figure S 1. Flow diagrams in the simplex S_4 in which peer punishment is used for $H = 1$ (panel (a)) and $H = 3$ (panel (b)). Stable fixed points are depicted with solid blue circles, while unstable fixed points are depicted with open blue circles. Arrows indicate the direction of evolution. In the interior of simplex S_4, the red surface divides the system into two basins of attraction in dependence on the initial conditions. And their percentages are depicted in the pie chart. The domain below the red surface is the cooperative basin of attraction, which corresponds to the red region in the pie chart. The rest of space is the basin of attraction for defection, which corresponds to the green region in the pie chart. Other parameters: $r = 3$, $c = 1$, $G = 5$, $\alpha = 1.0$, $\beta = 0.3$, and $\gamma = 0.05$.

In Fig. S1, we show the flow diagrams in the interior of the unit simplex S_4 for infinite well-mixed populations when peer punishment is used. We find that the results similar to our main text are obtained again. In dependence on the initial conditions, the system will evolve to either the state of full defectors (vertex D) or the coexistence state of cooperators and unconditional punishers (segment PK). In addition, for low $H = 1$ the cooperative basin of attraction occupies 62.3% of the whole strategy state space in S_4. While for high $H = 3$ the cooperative basin of attraction occupies 50.6% of the whole strategy state space. With these model parameters, we find that the cooperative basin of attraction occupies 55.8% of the whole strategy state space (the triangle PDC) when the strategy of conditional punisher is not introduced. This comparison result indicates that there exists the double-edged

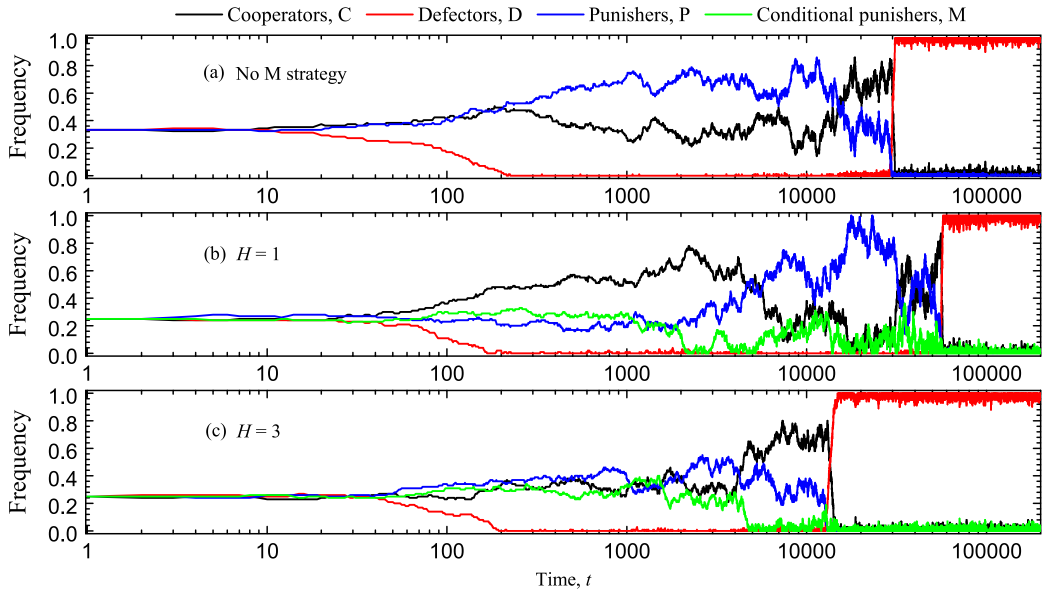

Figure S 2. Time evolution of strategies for three different situations with peer punishment. Panel (a) shows the time evolution of three strategies when conditional punishment is not considered. Panel (b) shows the time evolution of all four strategies for $H = 1$. Panel (c) shows the time evolution of all four strategies for $H = 3$. Individual-based simulations run over 10^9 time steps, and here we only show the outcomes for 2×10^5. Parameters: $r = 3$, $c = 1$, $G = 5$, $N = 100$, $\alpha = 1.0$, $\beta = 0.3$, $\gamma = 0.05$, $s = 2.0$, and $\mu = 0.001$.

sword effect induced by conditional punishment even when peer punishment is considered for infinite well-mixed populations.

Using a representative set of model parameters, we subsequently show the time evolution of strategies in finite well-mixed populations with mutation, as shown in Fig. S2. We respectively consider three different cases, and find the similar results to Fig. 3 shown in the main text. Specifically, the time duration of the quasi-stable state regarding the coexistence of cooperators and punishers in the case without the conditional punisher strategy is shorter than that in the case where a low threshold value is applied for conditional punishers. While it is longer than that in the case where a high threshold value is applied for conditional punishers. These results indicate that the double-edged sword effect induced by conditional punishers exists in finite well-mixed populations.

In Fig. S3, we present some series of representative snapshots under the regime of peer punishment. We can observe the same effect found in our main text that the conditional punishment shows the dual character. In comparison with the case without conditional punishers, if a low threshold value is applied, conditional punishers can be triggered to engage in the sanction easily. In this way, defectors only dominate over other strategists during the initial period of the evolution and propagate rapidly in the population. Once the cooperative players form the compact clusters, the direction of evolution will be reversed so that the population will evolve into the coexistence of cooperators and unconditional punishers. Otherwise, when the threshold value is high, conditional punishers cannot assist in resisting the invasion of defectors, and even offers some free-riding chance for defectors. The results imply that a double-edged sword effect induced by conditional punishment is also embodied in structured populations.

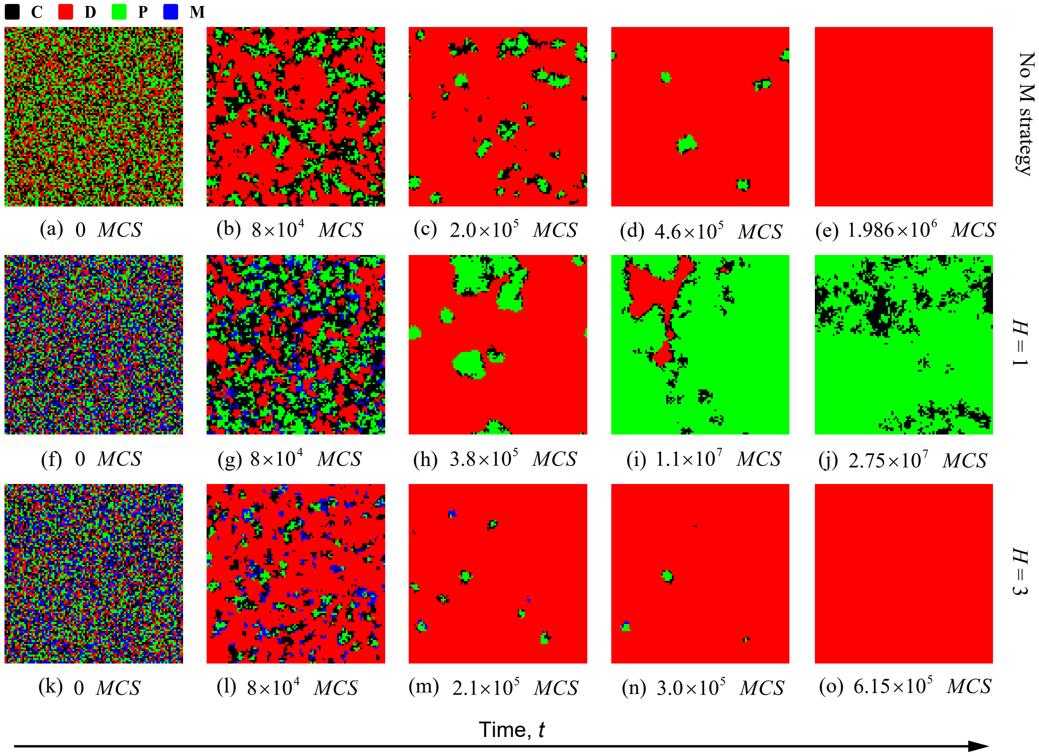

Figure S 3. Spatial patterns of strategies over time for three different situations with peer punishment. Cooperators (C) are denoted by black, defectors (D) by red, unconditional punishers (P) by green, and conditional punishers (M) by blue. Top row depicts the typical snapshots over time without the conditional punisher strategy. In the presence of conditional punishment, middle row depicts the typical snapshots over time for $H = 1$, whereas bottom row depicts the typical snapshots over time for $H = 3$. Parameters: $G = 5$, $r = 2.0$, $\alpha = 1.0$, $\beta = 0.49$, $c = 1.0$, $\gamma = 0.05$, $s = 2.0$, and $L = 100$.

1.3 Variant of peer punishment

When a variant of peer punishment [3] is used, we first show the flow diagrams in the interior of the unit simplex S_4 for infinite well-mixed populations, as shown in Fig. S4. Compared with the flow diagrams for the two aforementioned punishment regimes, we see that similar results are obtained again. The system will evolve to either the state of full defectors (vertex D) or to the coexistence of cooperators and unconditional punishers (segment PK), no matter whether the threshold value is low or high. In addition, even if the conditional punisher strategy is not considered in the variant of peer punishment [3], the system will still evolve to one of the two stable states, and the cooperative basin of attraction is 32.1% of the whole strategy state space (triangle PDC). Thus the cooperative basin of attraction is smaller than that in the case where conditional punishment is included in the public goods game and a low threshold $H = 1$ is applied (36.1%). But it is larger than that in the case where conditional punishment is included in the public goods game and a high threshold $H = 3$ is applied (29.5%). These results show that under the variant of peer punishment, the effect of conditional punishment on cooperation is still a double-edged sword.

In finite well-mixed populations, Fig. S5 shows the evolution of the competing strategies over time in three different cases with mutation. Although the homogeneous state of full defectors lasts for a long time during the whole evolutionary process, we can also observe the effect of a double-edged sword induced by conditional punishers. For a low threshold, conditional punishers have more opportunities to be triggered to engage in the sanction, which leads the population to experience a quasi-equilibrium state where defectors are suppressed. It displays the positive

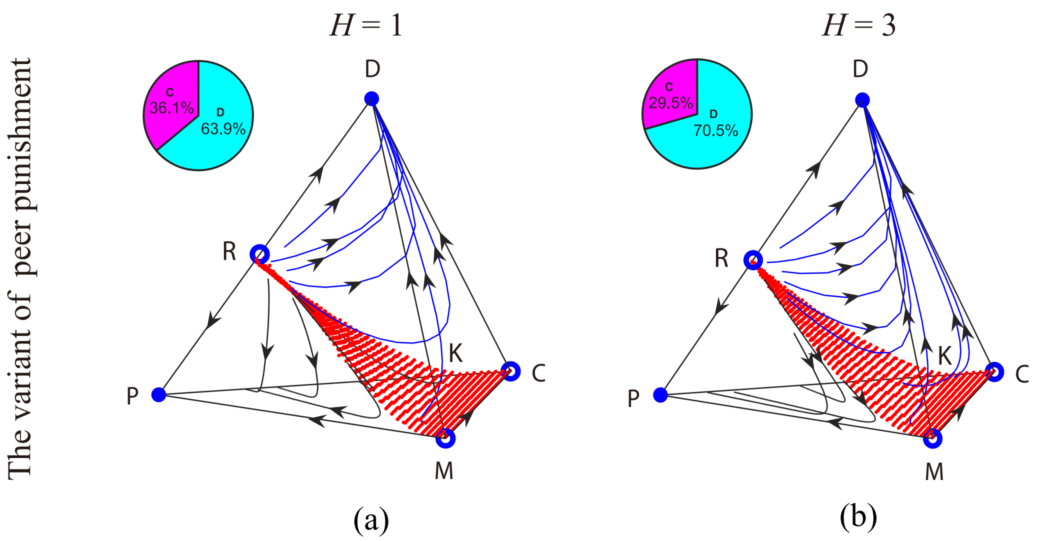

Figure S 4. Flow diagrams in the simplex S_4 in which the variant of peer punishment is used for $H = 1$ (panel (a)) and $H = 3$ (panel (b)). Stable fixed points are depicted with solid blue circles, while unstable fixed points are depicted with open blue circles. Arrows indicate the direction of evolution. In the interior of the simplex S_4, the red surface divides the system into two basins of attraction in dependence on the initial conditions. And their percentages are depicted in the pie chart. The domain below the red surface is the cooperative basin of attraction, corresponding to the red region in the pie chart. The rest of space is the basin of attraction for defection, corresponding to the green region in the pie chart. Other parameters: $r = 3$, $c = 1$, $G = 5$, $\alpha = 1.0$, $\beta = 0.7$, and $\gamma = 0.05$.

side of conditional punishers for improving the public cooperation. Otherwise, for a high threshold, they even provide more chances for defectors' free-riding such that the quasi-equilibrium does not emerge or lasts a fairly shorter time.

Finally, we present some series of representative snapshots in structured population for the variant of peer punishment, as shown in Fig. S6. When the threshold value is low, conditional punishers can be triggered to engage in the sanction easily, which helps cooperative individuals to form compact clusters (the second row in Fig. S6). Consequently, the direction of evolution is reversed, compared with the situation without conditional punishment where defectors take over the population finally (the first row in Fig. S6). However, because cooperators and conditional punishers both die out during the process of defectors' invasion, only unconditional punishers flourish in the population finally. On the contrary, when the threshold value is high, conditional punishers cannot assist in resisting the invasion of defectors, and even offers some free-riding chances for defectors. This can accelerate the demise of cooperators and unconditional punishers (the third row in Fig. S6). Therefore, the double-edged sword effect induced by conditional punishment can be still observed in structured populations.

2 Evolution of cooperation when anti-social punishment is considered

In addition to the universal concept that punishment is targeted at free-riders, the anti-social punishment that non-cooperators punish cooperators has received much attention [4, 5, 6, 7]. To include the possibility of anti-social punishment, here we consider a variant of our main model that defectors will retaliate all members in the group when they suffer from the sanction from the community of punishers [8]. Particularly, we are more interested in

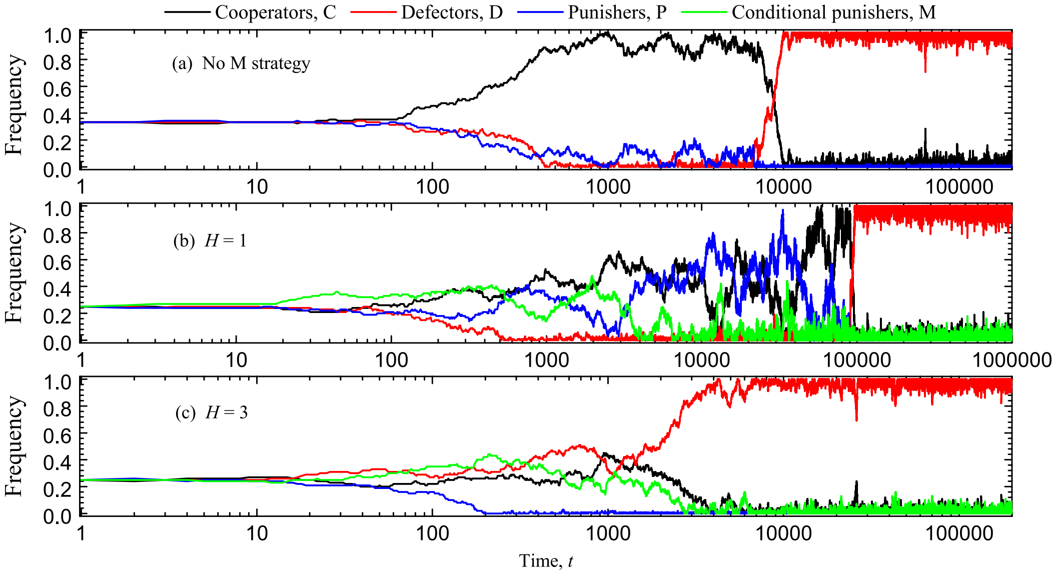

Figure S 5. Time evolution of strategies for three different situations with the variant of peer punishment. Panel (a) shows the time evolution of three strategies when conditional punishment is not considered. Panel (b) shows the time evolution of all four strategies for $H = 1$. Panel (c) shows the time evolution of all four strategies for $H = 3$. Individual-based simulations run over 10^9 time steps, and here we only show the outcomes for 10^5 – 10^6. Parameters: $r = 3$, $c = 1$, $G = 5$, $N = 100$, $\alpha = 0.7$, $\beta = 0.7$, $\gamma = 0.05$, $s = 2.0$, and $\mu = 0.001$.

the evolutionary dynamics in finite well-mixed populations, and whether cooperation can be still maintained in the presence of anti-social punishment.

Based on the punishment regime in our main text, the probability that a defector suffers from the sanction from the community of punishers is

$$\rho = 1 - \frac{\binom{N-Z-1}{G-1}}{\binom{N-1}{G-1}}, \tag{3}$$

where N, G, and Z are population size, group size, and the number of unconditional punishers in the population, respectively. Then, the average payoffs for pure cooperators (C), defectors (D), unconditional punishers (P), and conditional punishers (M) are given by

$$P'_C = P_C - \rho(G-1)\frac{Y}{N-1}\alpha, \tag{4}$$

$$P'_D = P_D - \rho(G-1)\beta, \tag{5}$$

$$P'_P = P_P - (G-1)\frac{Y}{N-1}\alpha, \tag{6}$$

and

$$P'_M = P_M - \rho(G-1)\frac{Y}{N-1}\alpha, \tag{7}$$

where P_C, P_D, P_P, and P_M are average payoffs for pure cooperators, defectors, unconditional punishers, and conditional punishers excluding the possibility of anti-social punishment, which are given by Eqs. (7)–(10) in the main text. And Y, α, and β denote the number of defectors, the fine imposed on each punished individual, and the cost of punishment, respectively.

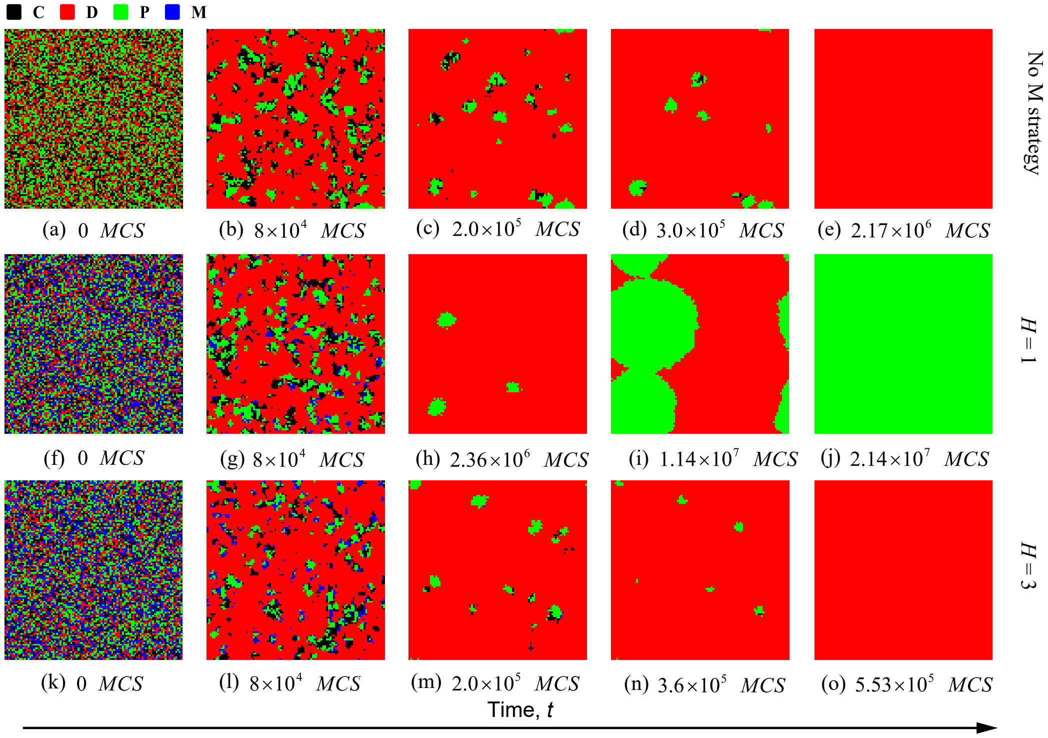

Figure S 6. Spatial patterns of strategies over time for three different situations when the variant of peer punishment is used. Cooperators (C) are denoted by black, defectors (D) by red, unconditional punishers (P) by green, and conditional punishers (M) by blue. Top row depicts the typical snapshots over time without the conditional punisher strategy. Middle row depicts the typical snapshots over time in the case where the strategy of conditional punisher is included in the public goods game and the threshold value is $H = 1$. Bottom row depicts the typical snapshots over time for $H = 3$ at which conditional punishment is considered. Parameters: $G = 5$, $r = 2.0$, $\alpha = 1.0$, $\beta = 0.25$, $c = 1.0$, $\gamma = 0.05$, $s = 2.0$, and $L = 100$.

By individual-based simulations in finite populations, Fig. S7 shows the time evolution of strategies in different mutation rates. Surprisingly, when anti-social punishment is considered, the state of full defectors is not evolutionarily stable any more. During the process of evolution, defectors are always at disadvantage. Accordingly, other cooperative strategies prevail in the population. Consequently, cooperation is sustained. Among these three cooperative strategies, due to neutral drift, cooperators and unconditional punishers dominate each other, alternately. In addition, because conditional punishers need to bear a fixed observation cost, pure cooperators and unconditional punishers are more prevalent than them. And this result is robust against small or moderate mutation rates. Particularly, by calculating the average frequencies of strategies, we find that the double-edged sword effect induced by conditional punishment is substantially weakened when anti-social punishment is considered (see Fig. S8). When conditional punishment is considered in the public goods game and a high threshold value is applied, similar to the previous findings [5, 7, 8], conditional punishment no longer promotes cooperation when anti-social punishment is possible. However, if a low threshold value is applied in the presence of conditional punishment, the positive side that conditional punishment promotes the evolution of cooperation is weakened in comparison with the case where anti-social punishment is excluded a priori (compare Fig. S8 with Fig.4 in the main text).

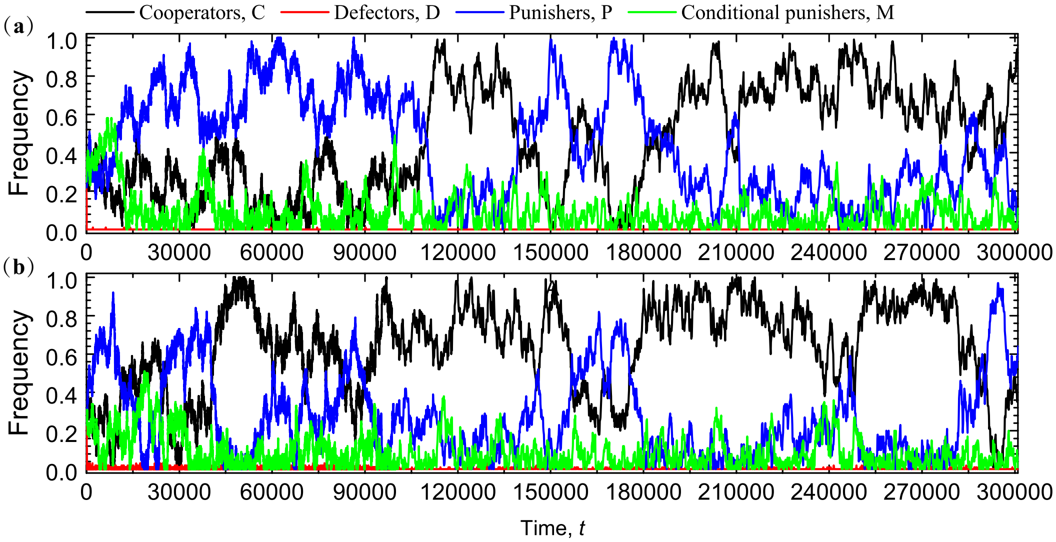

Figure S 7. Time evolution of strategies for different mutation rates when anti-social punishment is considered. Panel (a) shows the time evolution of strategies in the presence of conditional punishers and the mutation rate is $\mu = 0.001$, but $\mu = 0.1$ for panel (b). Other parameters: $r = 3$, $c = 1$, $G = 5$, $N = 100$, $\alpha = 1.0$, $\beta = 0.7$, $\gamma = 0.05$, $s = 2.0$, and $H = 2$.

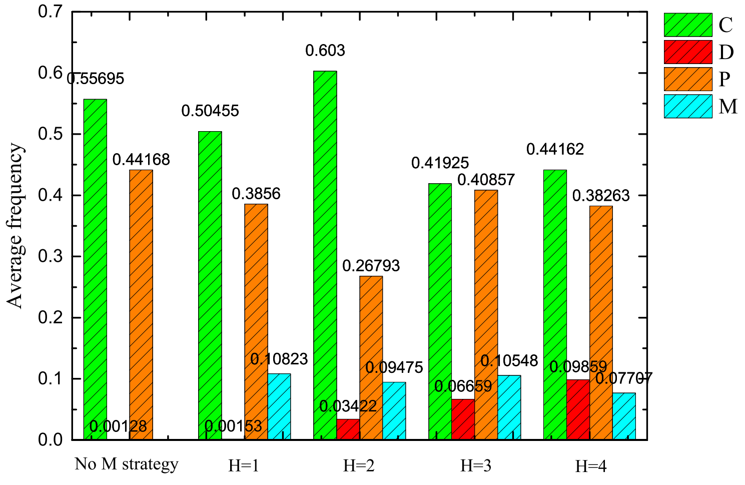

Figure S 8. Average frequencies of strategies when anti-social punishment is considered. When conditional punishment is considered in the public goods game and a high threshold value is applied ($H = 3$ or $H = 4$), conditional punishment no longer promotes cooperation. However, if a low threshold value is applied in the presence of conditional punishment ($H = 1$ or $H = 2$), the positive side that conditional punishment promotes the evolution of cooperation is weakened when anti-social punishment is possible. Individual-based simulations run over 10^7 time steps, and the average frequencies are obtained by averaging the last 10^4 time steps and by doing 30 independent runs. Parameters: $r = 3$, $c = 1$, $G = 5$, $N = 100$, $\alpha = 1.0$, $\beta = 0.7$, $\gamma = 0.05$, $s = 2.0$, and $\mu = 0.1$.

References

[1] K. Sigmund, H. De Silva, A. Traulsen, and C. Hauert. Social learning promotes institutions for governing the commons. *Nature*, 466(7308): 861–863, 2010.

[2] R. Boyd, H. Gintis, S. Bowles, and P. J Richerson. The evolution of altruistic punishment. *Proc. Natl. Acad. Sci. USA*, 100(6): 3531–3535, 2003.

[3] X. Chen, A. Szolnoki, and M. Perc. Probabilistic sharing solves the problem of costly punishment. *New J. Phys.*, 16(8): 083016, 2014.

[4] B. Herrmann, C. Thöni, and S. Gächter. Antisocial punishment across societies. *Science*, 319(5868):1362–1367, 2008.

[5] D. G. Rand and M. A. Nowak. The evolution of anti-social punishment in optional public goods games. *Nat. Commun.*, 2:434, 2011.

[6] C. Hilbe and A. Traulsen. Emergence of responsible sanctions without second order free riders, antisocial punishment or spite. *Sci. Rep.*, 2:458, 2012.

[7] O. P. Hauser, M. A. Nowak, and D. G. Rand. Punishment does not promote cooperation under exploration dynamics when anti-social punishment is possible. *J. Theor. Biol.*, 360:163–171, 2014.

[8] M. A. Janssen and C. Bushman. Evolution of cooperation and altruistic punishment when retaliation is possible. *J. Theor. Biol.*, 254(3):541–545, 2008.
